# Structural Characterization of Polysaccharides from *Dendrobium officinale* and Their Effects on Apoptosis of HeLa Cell Line

**DOI:** 10.3390/molecules23102484

**Published:** 2018-09-27

**Authors:** Wenxia Yu, Zhiyao Ren, Xiaofeng Zhang, Shangping Xing, Shengchang Tao, Chenxing Liu, Gang Wei, Yuan Yuan, Zhouxi Lei

**Affiliations:** 1 School of Pharmaceutical Sciences, Guangzhou University of Chinese Medicine, No. 232 Wai Huan Dong Road, Higher education mega center, Panyu District, Guangzhou 511400, China; yuwenxia3486@outlook.com (W.Y.); r._what@163.com (Z.R.); 15521076223@163.com (X.Z.); shaopingxing@gmail.com (S.X.); taoshengchang@hotmail.com (S.T.); liuchenxingstar@126.com (C.L.); 2 National Resource Center for Chinese Materia Medica, China Academy of Chinese Medical Sciences, Beijing 100000, China; 3 Guangdong Institute of Traditional Chinese Medicine, Guangzhou 510000, China

**Keywords:** *Dendrobium officinale*, structure elucidation, anti-tumor activity, plantation mode

## Abstract

*Dendrobium officinale* is a widely used medicinal plant in China with numerous bio-activities. However, the main structure and anti-tumor activity of the polysaccharides from this plant have not been investigated. In this study, we elucidated the main structure of polysaccharides purified with DEAE and Sephadex G-25 from *Dendrobium officinale* grown under different planting conditions. In addition, the anti-tumor activity was tested via MTT assays. The results showed that the polysaccharides of *Dendrobium officinale* grown under different conditions were almost the same, with slight differences in the branched chain; both polysaccharide fractions consisted of (1→4)-linked mannose and (1→4)-linked glucose, with an O-acetyl group in the mannose. After degradation, the polysaccharide fractions from wild plants showed significant anti-proliferation activity in HeLa cells. The fractions F1 and F3 induced apoptosis by up-regulating the expression of ERK, JNK, and p38. We concluded that polysaccharides from *Dendrobium officinale* planted in the wild exhibit significant anti-tumor effects only after being degraded to smaller molecular weight species. The planting mode is a significant factor in the pharmacological activity of *Dendrobium officinale*. We advise that the planting conditions for *Dendrobium officinale* should be changed.

## 1. Introduction

*Dendrobium officinale* is a medicinal plant that has been widely used in China for thousands of years. As the medicinal materials market has grown, the demand for the medicinal plant *Dendrobium officinale* has increased dramatically [1]. Since the wild resource is limited, researchers have developed a series of greenhouse cultivation methods to improve productivity. However, the metabolic components in plants are influenced greatly by the environment. Plants planted in the greenhouse are different from those grown in the wild. Based on our observation, the external properties of this medicinal plant planted in the wild and cultivated in the greenhouse are different. However, no studies have reported pharmacological differences between these two planting modes. Modern pharmacological studies have revealed that the stem extract of *Dendrobium officinale* exhibits significant bio-activities, including anti-inflammatory [2], anti-oxidant [3], anti-tumor [4], salivary secretion promoting [5], and immunomodulatory [6] activities. A study of the bio-active ingredients in *Dendrobium officinale* showed that several types of active compounds were present in this medicinal plant, including alkaloids, flavonoids, phenanthrenes, and polysaccharides [1]. Polysaccharides are the main component and account for 20% to 40% of the total compounds [7]. In recent years, several studies on the structure of polysaccharides as well as the bio-activities of *Dendrobium* species have been reported [8,9,10,11,12,13,14,15,16,17,18]. However, a literature investigation showed that a study of the anti-tumor activities of the polysaccharides in *Dendrobium officinale* was still missing. No papers have reported the structure and anti-tumor activity differences in polysaccharides from *Dendrobium officinale* grown under different conditions. In this study, we compared the structure differences in polysaccharides from *Dendrobium officinale* grown under two different conditions, and their anti-tumor properties were also investigated. 

## 2. Results

### 2.1. Elution Graph of the Purified Polysaccharide Fractions (PPFs)

**Figure 1:**
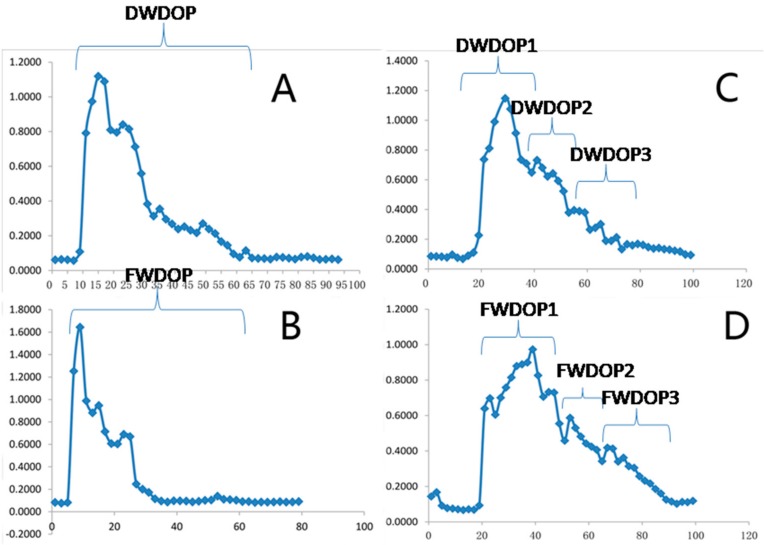
The (**A**,**B**) DEAE and (**C**,**D**) Sephadex G-25 elution graphs of DWDOP and FWDOP.

After deproteinization with Sevag reagents, the crude polysaccharides were purified with a DEAE column and a Sephadex G-25 column. The elution graph is shown in Figure 1. All the tubes with polysaccharides were collected and concentrated. According to the elution graph of the Sephadex G-25 column, the PPFs from the two sources of *Dendrobium officinale* were divided into two groups: the first group was named DWDOP and the second, FWDOP. The DWDOP represented PPFs from *Dendrobium officinale* planted in the greenhouse, whereas the FWDOP represented PPFs from plants grown in the wild. Each group obtained three polysaccharide fractions; these purified polysaccharide fractions were named DWDOP1, DWDOP2, and DWOPD3 and FWDOP1, FWDOP2, and FWDOP3, respectively. From the graph, DWDOP1 and FWDOP1 were the two major purified polysaccharide fractions. Therefore, DWDOP1 and FWDOP1 were further analyzed for structural elucidation. 

### 2.2. Molecular Weight and Monosaccharide Composition of the PPFs

**Figure 2:**
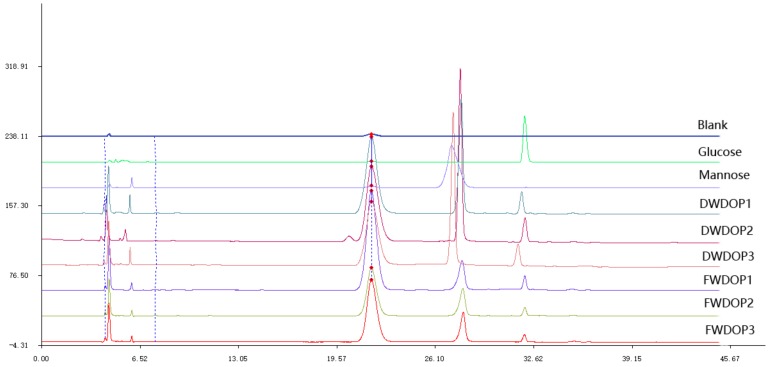
The HPLC of blank control solution, glucose standard solution, mannose standard solution and pure polysaccharides fractions DWDOP1, DWDOP2, DWDOP3, FWDOP1, FWDOP2, FWDOP3.

From the HPLC graph of the monosaccharide analysis, all six PPFs consisted of mannose and glucose. The mannose content in all PPFs was higher than the glucose content. The mannose/glucose ratio in DWDOP1, DWDOP2, and DWDOP3 and FWDOP1, FWDOP2, and FWDOP3 was 6.76, 6.09, and 4.50 and 7.46, 6.85, and 6.84, respectively. The monosaccharide analysis graph is shown in Figure 2. The molecular weight of the PPFs was determined by high performance gel permeation chromatography (HPGPC) using a series of dextran standard references. The exact molecular weight of the dextran standard and the retention time in HPGPC were used for establishing a standard curve. The equation of the standard curve calculated using the dextran standards is shown below:

Y = −1.9145X + 27.106(1)

R^2^ = 0.9929 (2)

The molecular weight of the four pure polysaccharide fractions, specifically, FWDOP1, FWDOP2, DWDOP1, and DWDOP2, was 1,341,061 Da, 540,216 Da, 1,415,641 Da, and 1,320,256 Da. Both DWDOP3 and FWDOP3 were not pure fractions. 

### 2.3. Structure of DWDOP1 and FWDOP1

**Figure 3:**
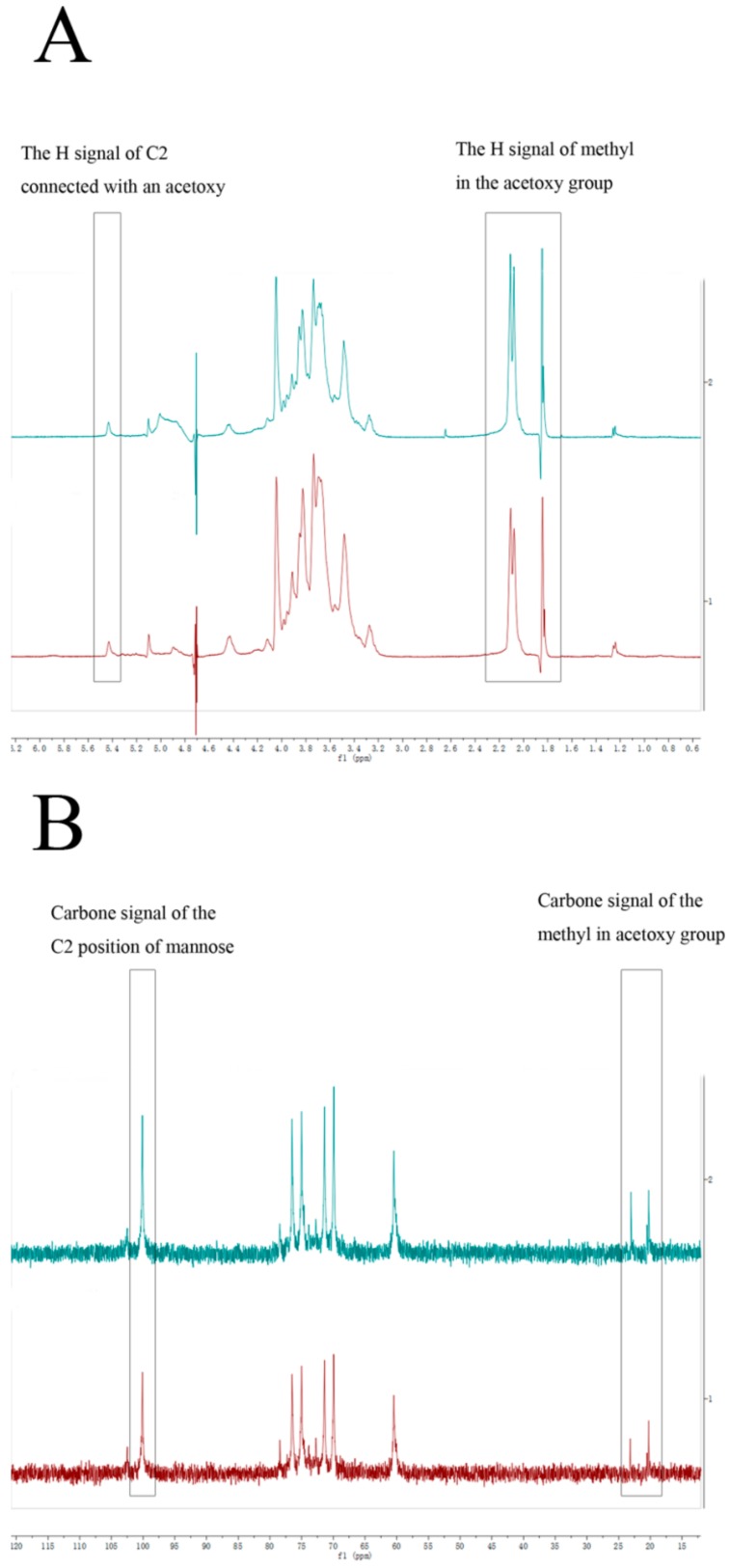
The (**A**) ^1^H-NMR spectra of DWDOP1 and FWDOP1; the (**B**) ^13^C spectra of DWDOP1 and FWDOP1.

The structure of DWDOP1 and FWDOP1 was elucidated through 1D and 2D-NMR analysis. The ^1^H-NMR signals for DWDOP1 and FWDOP1 were almost the same; DWDOP1 signals were observed at δ5.43, 5.10, 4.89, 4.42, 4.11, 4.04, 3.98, 3.95, 3.91, 3.85, 3.83, 3.74, 3.70, 3.56, 3.48, 3.28, 2.11, 2.08, and 1.84. FWDOP1 signals were located at δ5.44, 5.10, 5.02, 4.43, 4.05, 3.98, 3.96, 3.92, 3.88, 3.86, 3.83, 3.74, 3.69, 3.67, 3.56, 3.49, 3.27, 2.11, 2.08, and 1.85. From the ^1^H-NMR signals, a signal at 5.43 or 5.44 is observed in the spectrum of both polysaccharides. This signal is unique and represents the hydrogen in the skeleton of a sugar connected to an acetoxy group. In addition, the signals from 1.84 to 2.11 should be assigned to the hydrogen of the methyl of the acetoxy group. It can be speculated that both DWDOP1 and FWDOP1 contain an acetoxy group. The ^13^C-NMR signals for DWDOP1 and FWDOP1 were also almost the same. For DWDOP1, signals in the^13^C-NMR spectrum were located at δ102.48, 100.12, 78.45, 76.51, 75.00, 73.84, 72.76, 71.40, 69.92, 60.47, 23.04, and 20.52. The FWDOP1 ^13^C-NMR spectrum showed signals at δ102.48, 100.11, 78.43, 76.49, 75.00, 73.86, 72.74, 71.40, 69.93, 60.45, 23.17, and 20.53. From these spectra, 102.48 should be assigned to the carbon at the 2 position of the mannose connected with the acetoxy group. The signals from 20.52 to 23.17 should be assigned to the methyl carbon of the acetoxy group. The 1D-NMR spectra are shown in Figure 3.

**Figure 4:**
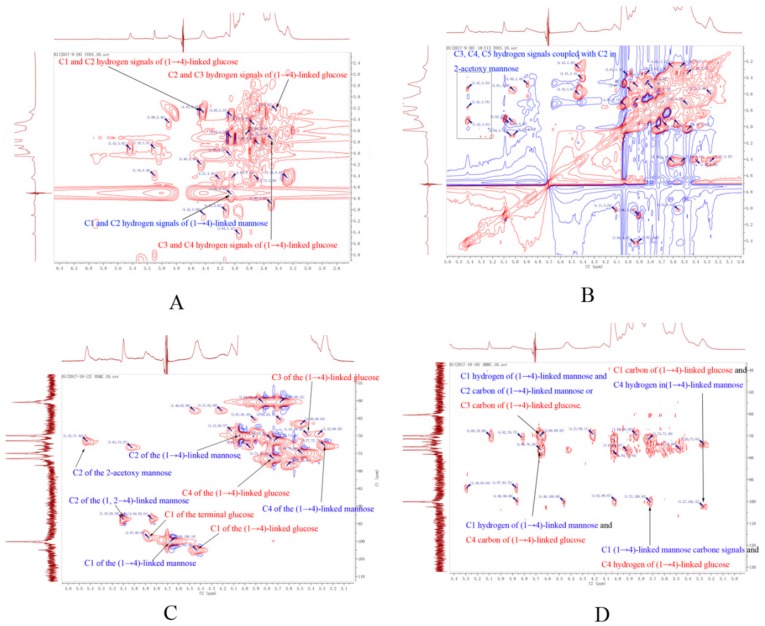
The 2D-NMR spectroscopy signals of (**A**) ^1^H-^1^H COSY, (**B**) ^1^H-^1^H TOSY, (**C**) ^1^H-^13^C HSQC, and (**D**) ^1^H-^13^C HMBC.

**Figure 5:**
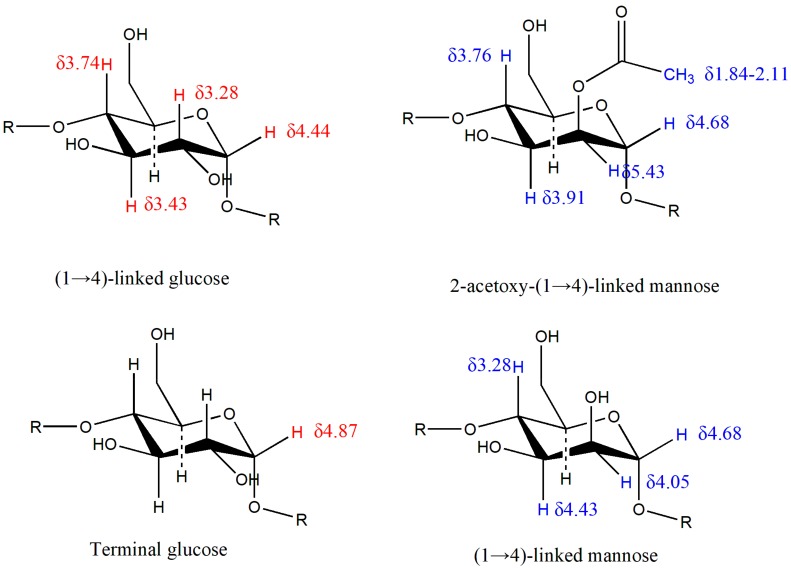
The attributed chemical shifts of all atoms.

Apart from 1D-NMR spectroscopy, 2D-NMR spectroscopy (^1^H-^1^H COSY, TOSY, ^13^C-^1^H HSQC, and ^13^C-^1^H HMBC) was also applied. From the 1D and 2D-NMR spectra, we can speculate on the structure of the DWDOP1 and FWDOP1 compounds. The annotation of the 2D-NMR signals is shown in Figure 4. The attribution of the NMR signals was based on published papers and structural elucidation experience [19,20,21,22]. First, we characterized the structure of DWDOP1. From the HSQC signals, we found the (71.46, 5.43) signal, which should be attributed to C_2_ of 2-acetoxy-(1→4)-linked mannose. The signals (102.46, 4.44) and (100.10, 4.68) should be attributed to coupling between the anomer carbon and hydrogen in (1→4)-linked glucose and (1→4)-linked mannose, respectively. From the COSY signals, we found the signals (4.43, 3.28), (3.23, 3.43) and (3.48, 3.74). The signal at δ4.43 represents the anomer hydrogen of (1→4)-linked glucose. Therefore, δ3.28 can be attributed to the C_2_ hydrogen of β-(1→4)-linked glucose. The δ3.28 and δ3.23, δ3.43, and δ3.48 signals were the same signals and were correlated with each other. Therefore, we can speculate that the δ3.43 signal represents the C_3_ hydrogen of (1→4)-linked glucose, whereas δ3.74 can be attributed to the C_4_ hydrogen of (1→4)-linked glucose. From the HSQC spectrum, we found the signal (76.49, 3.74). The signal 76.49 should be attributed to the C4 carbon of (1→4)-linked glucose. Searching the HMBC spectrum, we found the signal (100.04, 3.72); the signal 100.04 is the same as the 100.10 signal of the anomer carbon of (1→4)-linked mannose. Therefore, we can confirm that C4 of (1→4)-linked glucose is connected to C1 of (1→4)-linked mannose. In the COSY signals, a (4.73, 4.05) correlation was observed; the signal δ4.73 was close to the δ4.68 signal, and thus, δ4.73 should be attributed to the anomer hydrogen of (1→4)-linked mannose, and δ4.05 should be attributed to the C2 hydrogen of (1→4)-linked mannose. The signal (4.04, 4.43) was the coupling signal between the C2 hydrogen and C3 hydrogen of (1→4)-linked mannose, and the signal (4.43, 3.28) was the coupling signal of the C3 and C4 hydrogen signal. Therefore, we can confirm that the signal δ3.28 is the signal of the C4 hydrogen in (1→4)-linked mannose. From the HSQC spectrum, we found the signal (72.79, 3.27) and can confirm that 72.29 represents the C4 carbon of (1→4)-linked mannose. From the HMBC spectrum, we found the signal (3.27, 102.42). We had previously confirmed that 3.27 should be attributed to the C4 hydrogen and 102.42 should be attributed to the anomer carbon of (1→4)-linked glucose. From here, we can confirm that C1 of (1→4)-linked glucose is connected with C4 of the (1→4)-linked mannose. The connection of (1→4)-linked glucose and (1→4)-linked mannose constructs the skeleton of the DWDOP1 compound. From the 1D-NMR spectra, we confirmed that 5.42 represented the 2-acetoxy-(1→4)-linked mannose hydrogen. In the TOSY spectrum, we confirmed that 3.91, 3.76, and 3.53 indicated the hydrogen of C3, C4, and C5, and that 4.83 is the anomer hydrogen signal. In the HMBC spectrum, we found the signal (100.04, 3.72). This signal may also be attributed to (1→4)-linked mannose connected with 2-acetoxy-(1→4)-linked mannose. In the HMBC spectrum, we found the signals (4.66, 76.20) and (4.66, 69.74). The former signal should be attributed to the coupling signal of the anomer hydrogen of (1→4)-linked mannose and the C4 carbon of (1→4)-linked glucose. The latter indicated that the anomer hydrogen of mannose was coupled with another carbon. In the HSQC spectrum, the signals (69.93, 4.04) and (69.58, 3.43) were observed. The signals 4.04 and 3.43 were from the C2 and C3 position hydrogen of (1→4)-linked mannose and (1→4)-linked glucose. Therefore, we speculated that the signal (4.66, 69.74) in the HMBC spectrum was the coupling signal of the anomer hydrogen of (1→4)-linked mannose and the C2 carbon of (1→4)-linked mannose or the C3 carbon of (1→4)-linked glucose. In the HSQC spectrum, we also found the signals (93.58, 5.10) and (99.03, 4.87). The (99.03, 4.87) signal should be attributed to the anomer of the terminal glucose, and (93.58, 5.10) may be the C2 hydrogen signal of (1, 2→4)-linked mannose. Here, we can speculate that the skeleton of the polysaccharides in DWDOP1 mainly consisted of (1→4)-linked mannose connected with (1→4)-linked glucose. The chemical shifts of all atoms are shown in Figure 5. The complete NMR figures of all polysaccharides can be reached in the supplemental data.

The structure of the FWDOP1 polysaccharide was almost the same as that of the DWDOP1 polysaccharide, mainly consisting of (1→4)-linked mannose and (1→4)-linked glucose, 2-acetoxy-(1→4)-linked mannose. The primary difference between these two polysaccharides was based on the three characterized signals in the HSQC spectra, namely, (90.52, 4.06), (88.40, 4.03), and (80.70, 4.69). The carbon signals from 80 to 88 were the characteristic signals of the furan configuration of sugar [23]. The hydrogen signal 4.03 should be attributed to the C2 hydrogen of mannose, whereas 4.69 should be attributed to the anomer hydrogen of mannose. Therefore, we speculated that a mannofuranose was present in FWDOP1. 

**Figure 6:**
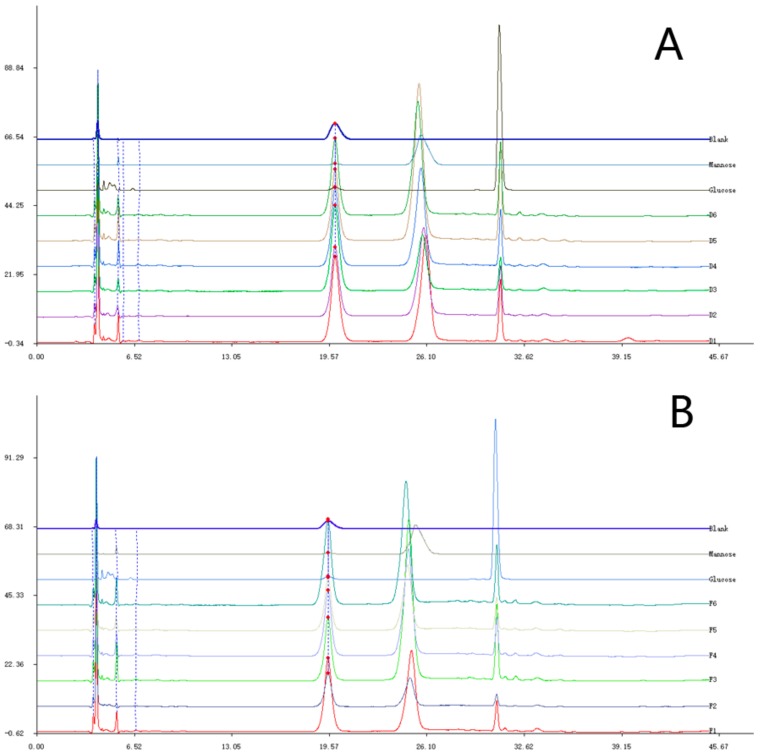
Monosaccharide analysis of (**A**) D1–D6 and (**B**) F1–F6.

Based on the molecular weight and the monosaccharide composition of DPFs after degradation, a DPF series was obtained. The degraded DPFs from *Dendrobium officinale* planted in a greenhouse were named D1–D6. The degraded DPFs from the plants planted in the wild were named F1–F6. The molecular weight of the DPFs was calculated with a standard curve constructed using a series of standard references: T5, T11, T80, T273, and T667. The standard curve used was the same as the one described in the previous section. After calculation, the molecular weight of D1 to D6 was 119,374 Da, 83,162 Da, 60,532 Da, 44,532 Da, 40,313 Da, and 21,818 Da. The molecular weight of F1 to F6 was 86,993 Da, 75,819 Da, 62,872 Da, 57,798 Da, 24,188 Da, and 29,904 Da. From the HPLC spectra, the monosaccharide composition of the DPFs was different. All DPFs consisted of mannose and glucose, and the ratio of mannose and glucose in D1 to D6 was 5.4, 5.7, 5.8, 5.7, 5.4, and 5.0, whereas the ratio in F1 to F6 was 8.0, 8.2, 6.6, 8.5, 6.2, and 6.5. The graph of HPLC was shown in Figure 6. From the results, the mannose content in F1 to F6 was larger than the content in D1 to D6. 

### 2.4. Effects of the DPFs on HeLa Cell Proliferation

**Figure 7:**
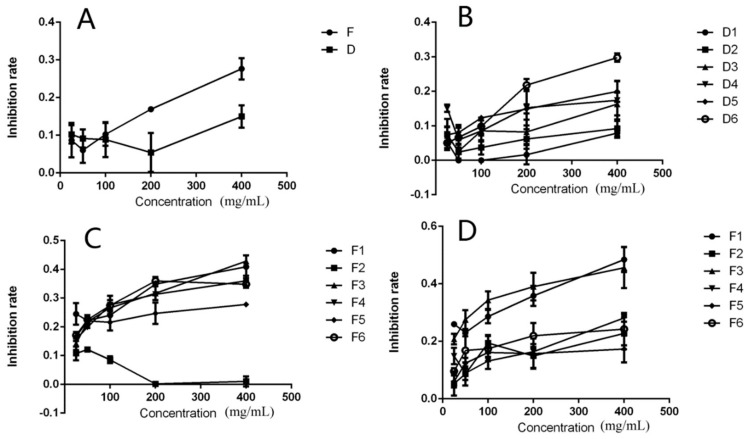
The anti-proliferation effects of (**A**) crude polysaccharides, (**B**) D1–D6, and (**C**) F1–F6 on HeLa cells after treatment for 24 h and (**D**) 48 h.

In this study, we applied MTT assays to detect the anti-proliferation activity of the CP and DPFs. From the graph, the CP from *Dendrobium officinale* planted in a green house or planted in the wild showed no significant activity on HeLa cells after treatment for 24 h. After treatment for 24 h at the highest concentration (400 μg/mL), the anti-proliferation activity of D1–D6 was 7.9%, 9.2%, 16.3%, 17.4%, 20.0% and 29.8%. D1–D6 exhibited no significant anti-proliferation activity in HeLa cells. The anti-proliferation activity of F1–F6 was 42.5%, 2.2%, 43.0%, 36.8%, 28.2%, and 35.9%. The DPFs F1 and F3 were the most effective. The anti-proliferation activity of F1 and F3 at concentrations ranging from 25 μg/mL to 400 μg/mL was 24.4%, 22.5%, 24.4%, 35.6%, and 42.5% and 15.2%, 21.7%, 27.0%, 31.6%, and 43.0%, respectively. The DPFs F1 and F3 exhibited significant anti-proliferation activity in a dose-dependent manner. We proceeded to further study the anti-proliferation activity of F1–F6. The activity of both F1 and F3 was high. When the concentration was 400 μg/mL, the anti-proliferation rate induced by F1 and F3 was 48.3% and 48%, respectively. For the other four DPFs (i.e., F2, F4, F5, and F6), the rate was 21.5%, 27.6%, 17.5%, and 25.2%, respectively. The graph of inhibition rate was shown in Figure 7. 

### 2.5. Apoptosis Rate of HeLa Cells after Treatment with F1 and F3 for 24 h and 48 h

**Figure 8:**
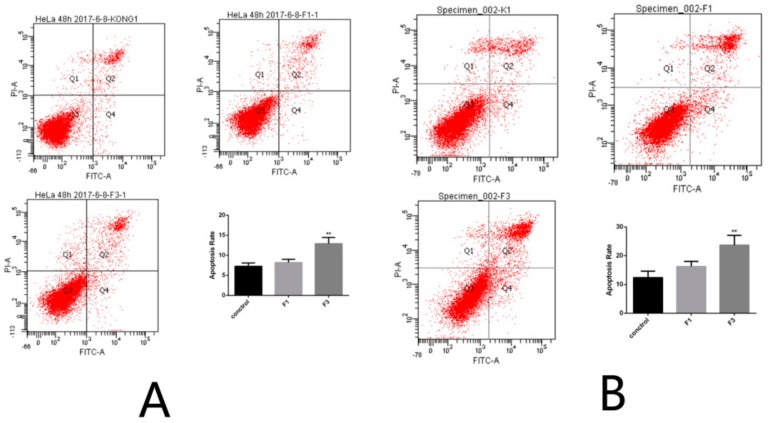
The apoptosis rate of HeLa cells after treatment with F1 and F3 for (**A**) 24 h and (**B**) 48 h.

The apoptosis rate induced by the DPFs F1 and F3 was tested using flow cytometry. After treatment with F1 and F3 for 24 h at a concentration of 400 μg/mL, the necrosis rate and percentage of HeLa cells in early and late phase apoptosis was 3.5%, 6.9%, and 1.3% and 2.4%, 9.9%, and 3.0%, respectively, which is statistically higher than in the control group treated with PBS. If the duration time was increased to 48 h with the same concentration of 400 μg/mL, the necrosis rate and percentage of HeLa cells in early phase and late phase apoptosis was 2.4%, 16.5%, and 7.2% and 12.3%, 16.3%, and 23.7%, respectively. The graph of flow cytometry was shown in Figure 8. The apoptosis rate of cells treated with F1 and F3 was significantly higher than that of control group cells. The results demonstrated that the DPFs F1 and F3 can induce apoptosis in HeLa cells. 

### 2.6. Morphology Changes in HeLa Cells after Treatment with F1 and F3 for 24 h and 48 h

**Figure 9:**
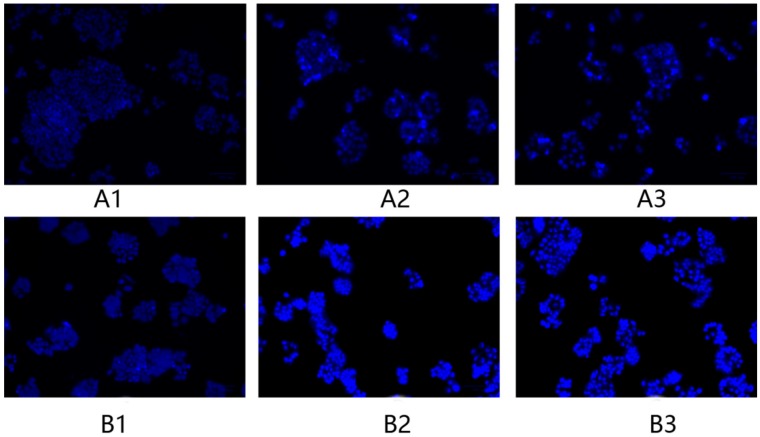
The morphology changes in HeLa cells in the (**A1**,**B1**) control group, (**A2**,**B2**) F1 treatment group, and (**A3**,**B3**) F3 treatment group after treatment for (**A**) 24 h and (**B**) 48 h.

The morphology changes in HeLa cells after treatment with F1 and F3 at a concentration of 400 μg/mL for 24 and 48 h was observed. As it was shown in Figure 9 of the microscopy images of cell morphology, no fluorescent dots were present in the control group of HeLa cells. However, in the F1- and F3-treated groups, some blue fluorescence was observed, which was caused by leakage of cellular content. In addition, after treatment for 48 h, the amount of blue fluorescence increased. As the treatment duration was increased, the morphology changes in the HeLa cells were exacerbated. 

### 2.7. Relative Expression of Apoptosis Genes and Proteins

**Figure 10:**
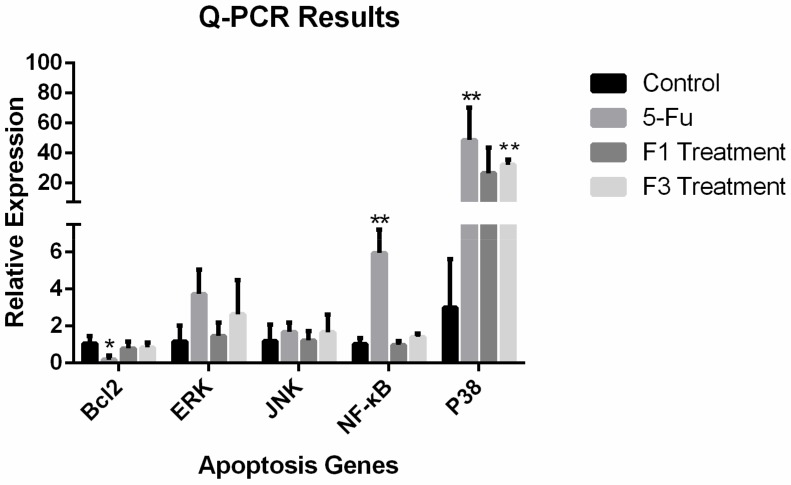
The relative expression level of apoptosis-related genes. (comparing with control group, * *p* ≤ 0.05; ** *p* ≤ 0.01).

**Figure 11:**
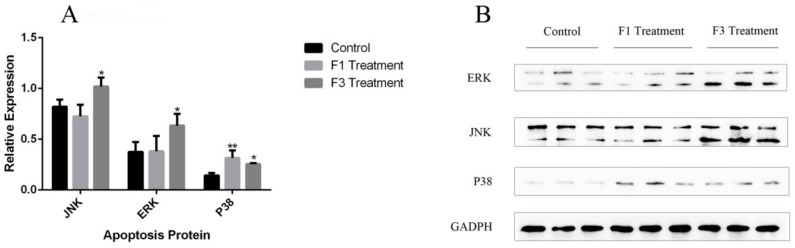
The relative expression level of apoptosis-related proteins (**A**) (comparing with control group, * *p* ≤ 0.05; ** *p* ≤ 0.01); (**B**) The graph of Westen-blot.

In this study, the relative expression level of five apoptosis-related genes, namely, Bcl-2, ERK, JNK, NF-κB, and p38, were tested. In the F1 and F3 group, the expression level of four of the five apoptosis-related genes in HeLa cells after treatment with 400 μg/mL F1 and F3 for 48 h showed no significant differences compared with the control group. However, the expression level of p38 in the F3 group was significantly higher than in the control group. F3 up-regulated the expression of the p38 gene in HeLa cells. In the positive drug group, three apoptosis-related genes, Bcl-2, NF-κB and p38, showed significant differences compared with the control group. The positive drug 5-FU can down-regulate the expression level of Bcl-2 and up-regulate the expression level of NF-κB and p38. The graph of relative expression of all tested genes was shown in Figure 10. Apart from the apoptosis-related genes, we also applied western blotting to test the expression of the apoptosis-related proteins JNK, ERK, and p38. From the graph of the western-blot results, and after treatment with F1 and F3, the protein expression of JNK, ERK, and p38 was significantly up-regulated compared with the control group. The graph of relative expression of tested protein was shown in Figure 11A, and the graph of Western-blot was shown in Figure 11B. 

## 3. Discussion

The plant *Dendrobium officinale* has been used for thousands of years in China as a folk medicine. In recent decades, scientists have found that the polysaccharides in this plant exhibit fine bio-activities, including anti-tumor, immunomodulatory, hypoglycemic, anti-inflammatory, and antioxidant activities. However, no studies have examined the structure of polysaccharides from *Dendrobium officinale* grown under different conditions or their anti-tumor activities. The results of this study have shown that the skeleton of the polysaccharides from wild-grown and greenhouse-grown *Dendrobium officinale* was the same and consisted of (1→4)-linked mannose and (1→4)-linked glucose. Differences were found in the mannose and glucose ratio, as well as in the side chains. The polysaccharides from wild plants may consist of the furan configuration of sugar, whereas this configuration was not present in the *Dendrobium officinale* planted in a greenhouse. The crude polysaccharides from both wild and greenhouse-grown plants exhibited no significant activities in HeLa cells. Through literature investigation, we found that molecular weight is a critical factor that influences the bio-activities of polysaccharides. Degradation by oxidative reagents can increase the activity of polysaccharides. In this study, the polysaccharides from wild plants exhibited certain anti-tumor activities in HeLa cells after degradation. It was obvious that molecular weight played an important role in anti-tumor activities. In addition, after degradation, the polysaccharide fractions from wild plants exhibited certain anti-tumor activity [24,25,26,27,28,29]. However, the activity of the polysaccharide fractions from greenhouse plants was still not significant. In addition, through monosaccharide analysis, we found that the mannose-related content in polysaccharide fractions from wild plants was significantly higher than in fractions from the greenhouse plants. Therefore, we speculated that the activity of polysaccharides from *Dendrobium officinale* was related to molecular weight as well as monosaccharide composition. We also speculated that the anti-tumor activities may be related to the furan configuration. In this study, we thoroughly investigated the anti-tumor mechanism of the degraded polysaccharide fractions. We found that the degraded polysaccharide fractions induced apoptosis in HeLa cells through the p38/MAPK signaling pathway. We believe that when people drink the water extract of *Dendrobium officinale*, the polysaccharide is degraded by the gastrointestinal tract before being absorbed into the blood. The degradation process in this study can be considered to mimic the gastrointestinal digestion process. Polysaccharides with a smaller molecular weight will be present after absorption. In this study, we compared the anti-tumor activities of polysaccharides from *Dendrobium officinale* grown under two different conditions and found that the anti-tumor effect of wild plants was significant. We therefore advise that the planting mode of *Dendrobium officinale* should be improved. Even though greenhouse cultivation can increase the production of this medicinal plant, simulating wild plant conditions is a better approach. 

## 4. Methods and Reagents

### 4.1. Materials and Reagents

The *Dendrobium officinale* plants used in this study were divided into two groups, those planted in the greenhouse and those planted in the wild. All plants were collected from Shaoguan City, Guangdong Province. After authentication by Gang Wei, professor at Guangdong University of Chinese Medicine, the plants were identified as *Dendrobium officinale*. 

The reagents used in the extraction, purification, and degradation are listed below. The DEAE column, Sephadex G-25 column, and PMP were purchased from Macklin (Macklin, Shanghai, China). T-series dextran was purchased from Sigma (Sigma-Aldrich, St. Louis, MO, USA). The reagents used in the in vitro experiments were Minimum Eagles’ Medium (MEM) (KeyGene, Jiangsu, China), Phosphate Buffer Saline (PBS) (KeyGene), fetal bovine serum, trypsin (KeyGene), a cell apoptosis kit array kit (KeyGene), 5-FU (Sigma-Aldrich), a Hoechst 33258 kit (KeyGene), Trizol (KeyGene), a cDNA synthesis kit (KeyGene), a qPCR reagent kit (abm Inc., New York, NY, USA), a protein extraction kit (KeyGene), an SDS-PAGE kit (KeyGene), an ECL kit (KeyGene), a BCA protein detection kit (KeyGene), a protein marker (KeyGene), a loading buffer (KeyGene), as well as GADPH (ABclonal, Wuhan, China), goat anti-mouse (ABclonal), and IgG antibodies (ABclonal). The HeLa cell line used in this study was purchased from iCell (iCell Bioscience Inc., Shanghai, China). 

### 4.2. Extraction of Dendrobium Officinale Polysaccharides

The stem of *Dendrobium officinale* was dried in an oven and crushed into powder before the experiments. Then, 50 g of the powder was weighed, 500 mL of petroleum ether was added to remove the lipids, and 500 mL of 80% ethanol was added to remove low polarity substances. The polysaccharide was extracted using 1500 mL of distilled water. The extract was precipitated with 80% ethanol to obtain the polysaccharides. The Sevag method was applied to remove protein in the polysaccharides to obtain crude polysaccharide (CP). 

### 4.3. Purification of Polysaccharides

The crude polysaccharide (CP) was re-dissolved in distilled water and purified with DEAE and Sephadex G-25 columns to obtain the purified polysaccharide fraction (PPF). The elution phase for the DEAE column was distilled water, the flow speed was 1 mL/min, and 100 tubes of the elution phase were collected. The elution phase for the Sephadex G-25 column was 0.2 M NaCl, the flow speed was set at 0.5 mL/min, and 100 tubes of the elution phase were collected. The polysaccharide content in the elution phase was tested using phenol-sulfuric acid. 

### 4.4. Oxidative Degradation of Crude Polysaccharides

The crude polysaccharide (CP) was re-dissolved in distilled water, a series of reagents were applied for degradation to obtain the degraded polysaccharide fractions (DPFs). The reagents used in this experiment were vitamin C, H_2_O_2_, FeCl_2_∙4H_2_O, and distilled water. Each of the final volumes of the degradation system was 50 mL. The degradation process was carried out in the dark, at room temperature (25 °C), and the duration time was 60 min. The ratios of these reagents used in the experiment are shown in Table 1. 

### 4.5. Detection of the Molecular Weight of PPF and DPF

An adequate amount of the PPF and DPF was weighed and dissolved in distilled water to a fixed concentration of 5 mg/mL to obtain sample solutions. T-series dextran was weighed precisely and dissolved in distilled water to obtain standard solutions. High Performance Gel Permeation Chromatography (HPGPC) was used in this study. The samples and the T-series dextran standards (MW: 667, 273, 147, 80, 11, and 5 kDa) were analyzed on an Agilent 1100 series HPLC system (Palo Alto, CA, USA) equipped with an RI-101SHODEX RID detector (Tosoh, Tokyo, Japan). The molecular weight of the samples was determined using a standard curve established with the standard solution. 

### 4.6. Detection of Monosaccharide Composition and Ratio of PPF and DPF

An adequate amount of the PPF and DPF was weighed and dissolved in distilled water to a fixed concentration of 1 mg/mL to obtain sample solutions. The standard solutions were prepared with glucose and mannose standard references using the same preparation method used for the sample solution. The blank control solution was also prepared with the same method without adding any substance. The experiment to detect the monosaccharide composition and ratio was performed as follows. First, the solution was hydrolyzed with HCl in an oven at 110 °C. Then, the pH value of the solution was adjusted to 7 with NaOH. PMP was added to the solution to react. The reaction was then subjected to monosaccharide detection using HPLC. The HPLC analysis was performed on an HPLC system (Shimadzu, Kyoto, Japan) with an XDB-C18 analytical column (4.6 × 150 mm, 5 μm, Agilent ZORBAX). The mobile phase was 0.05 M aqueous KH_2_PO_4_ (solvent A) and acetonitrile (solvent B).

### 4.7. NMR Spectrometer Analysis of PPFs

An amount of 30 mg PPF was weighed precisely and deuterium-exchanged by freeze drying three time from 99% D2O to obtain the sample solution. A 0.5 mL portion of the sample solution was transferred to a 5 mm tube. NMR spectra were recorded in 25 °C on a Bruker NMR spectrometer (Avance III HD 400 MHz Digital NMR Spectrometer, Bruker, Karlsruhe, Germany).

### 4.8. MTT Assay of HeLa Cells Treated with CP and DPF

The CP and DPF were dissolved in PBS at a fixed concentration of 4 mg/mL. The DPF solutions were filtered through 0.22-μm microporous filter membranes to avoid contamination. HeLa cells were cultured in MEM with 10% fetal bovine serum. The cells were transferred to a 96-well plate during exponential growth. After being cultured for 24 h, the cells were treated with DPFs in a concentration series for 24 h and 48 h. MTT was then added and incubated with the cells for 4 h. MTT was removed, and 100 μL of DMSO was added to each well. The absorption at 490 nm was tested using a microplate reader (BX51, Olympus Optical Co. Ltd., Tokyo, Japan). 

### 4.9. Detection of the Apoptosis Rate of HeLa Cells

The apoptosis rate of HeLa cells was tested by Annexin V-FITC/PI staining. The HeLa cells were seeded in 6-well plates during exponential growth and cultured for 24 h before being treated with DPFs. The cells were collected using EDT-free trypsin and washed with PBS three times. Then, the cells were re-suspended in 500 μL of binding buffer with 5 μL of Annexin V-FITC, PI was added into the cell suspension, and the cells were incubated in the dark at room temperature for 10 min. The apoptosis rate of the cells was tested using flow cytometry (Attune^®^ Acoustic Focusing Cytometer, Thermo Fisher Co. Ltd., Waltham, MA, USA). 

### 4.10. Morphological Observation of HeLa Cells

The morphology of the HeLa cells was observed with a fluorescence microscope after staining with Hoechst 33258, according to the following procedure. The HeLa cells were seeded in 6-well plates during exponential growth. The cells were cultured for 24 h before being treated with DPFs and incubated for 48 h. The upper MEM was removed, and the cells were washed with PBS three times. Then, 4% paraformaldehyde was added for fixation and incubated with the cells at 4 °C for 10 min. The 4% paraformaldehyde was removed, and the cells were washed three times with PBS. Hoechst 33258 (500 μL) was added to each well and incubated with the cells in dark conditions for 10 min. The Hoechst 33258 was removed, and the cells were washed two times with PBS. The morphology of the HeLa cells was observed using a fluorescence microscope (BX51, Olympus Optical Co. Ltd.). 

### 4.11. Q-PCR Assay of the Expression of Apoptosis-Related Genes

After treatment with DPFs and incubation for 48 h, RNA was extracted from the cells using an RNA extraction kit reagent. The concentration and quality of the extracted RNA was tested using a nucleic acid analyzer. Before proceeding to Q-PCR detection, cDNA was synthesized from the extracted RNA using cDNA synthesis kit reagents. The expression level of the apoptosis-related genes NF-κB, Bcl-2, p38, ERK, and JNK was determined by Q-PCR equipment (CFX96 Touch™ Real-Time PCR Detection System, Bio-rad Laboratories, Inc., Hercules, CA, USA). 

### 4.12. Western-Blotting Assay of MAPK Protein

After treatment with DPFs and incubation for 48 h, protein was extracted from the cells using lysing reagents. A BCA protein quantitative method was used to quantify the concentration of each protein sample. Then, the protein was separated by SDS-PAGE. The gel was selected after comparison with the protein marker, and the protein was transferred to a membrane. A blocking reagent was used to bind the protein before incubation with primary and secondary antibodies. The protein bands were developed using an ECL kit. The bands were recorded using a chemiluminescence analyzer (ChemiDoc™ MP system, Bio-rad Laboratories, Inc.), and the gray value was counted. 

## Figures and Tables

**Table 1:** The condition of degradation method.

Order	Vitamin C (mL)	H_2_O_2_ (mL)	FeCl_2_∙4H_2_O (mL)	CP	Water (mL)
1	0.5	0.05	0.05	30	19.4
2	1	0.1	0.1	30	18.8
3	2	0.2	0.2	30	17.6
4	4	0.4	0.4	30	15.2
5	5	0.5	0.5	30	14
6	6	0.6	0.6	30	12.6

## Supplementary Materials

The following are available online.
