# Supplementary material for: Structural Characterization of Polysaccharides from Dendrobium officinale and Their Effects on Apoptosis of HeLa Cell Line
Source: Molecules. 2018 Sep 27;23(10):2484. doi: 10.3390/molecules23102484 (PMC6222635; doi:10.3390/molecules23102484)
Supplement: Supplementary file 1 [file molecules-23-02484-s001.pdf]

# Supplement Data

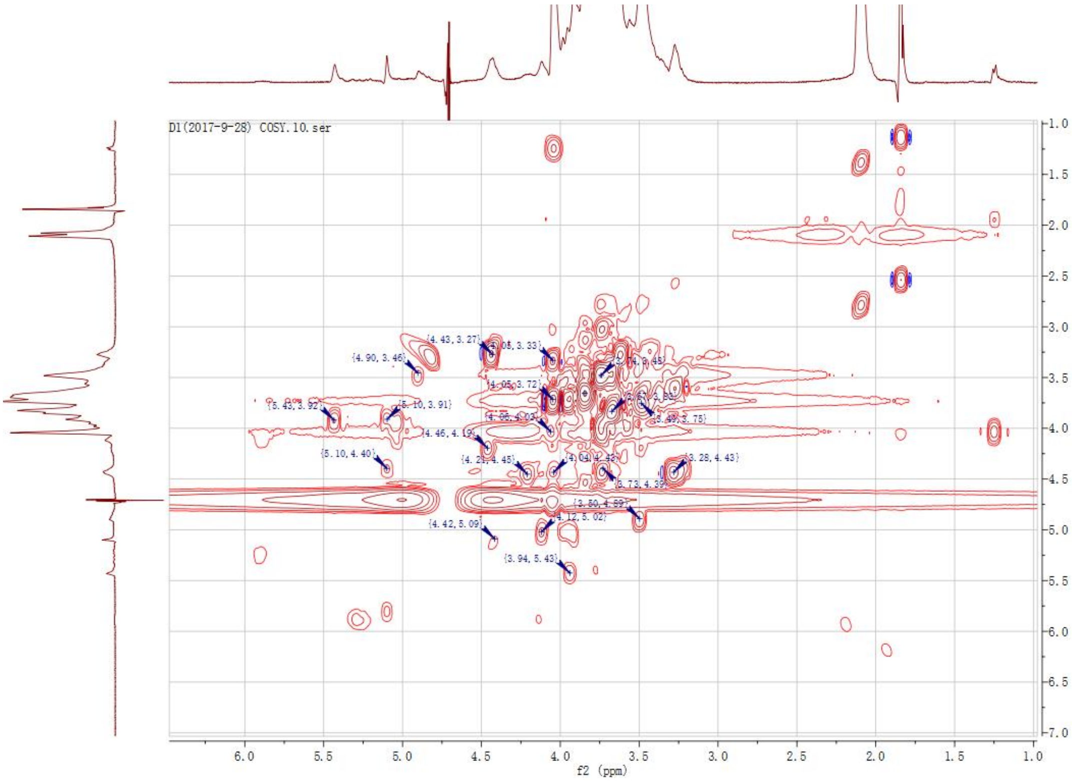

Figure S1 The $^{1}H$-$^{1}H$ COSY of DWDOP1

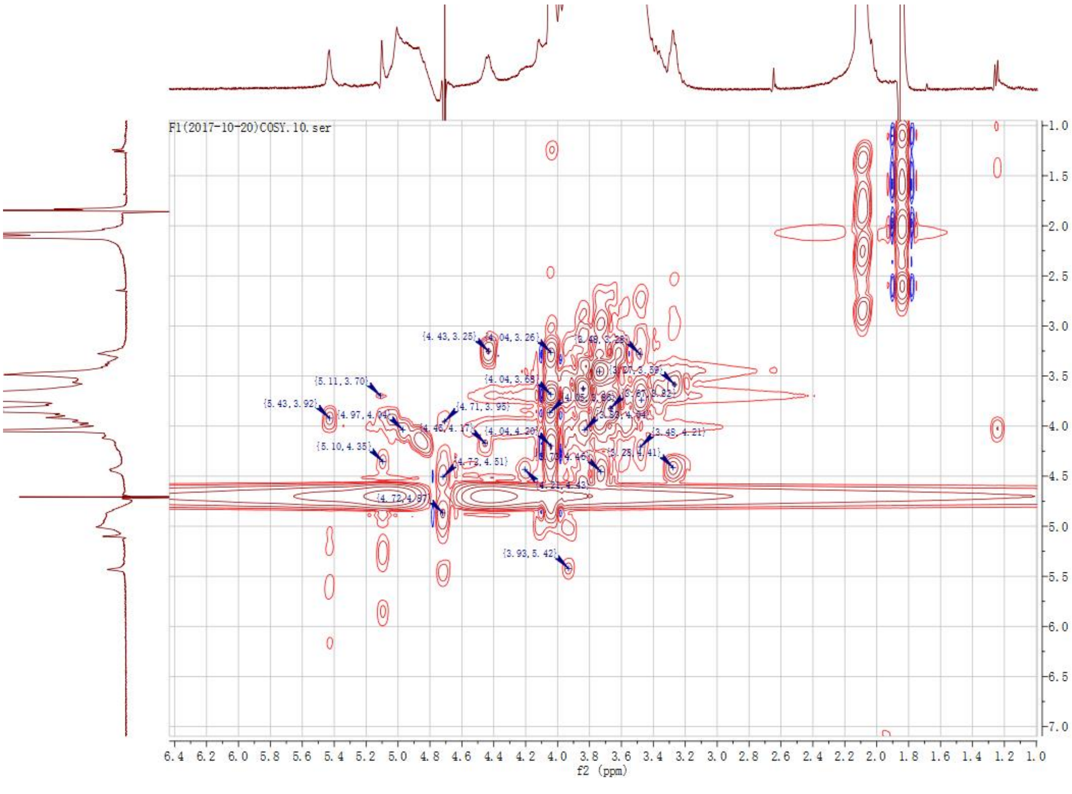

Figure S2 The $^{1}H$-$^{1}H$ COSY of FWDOP1

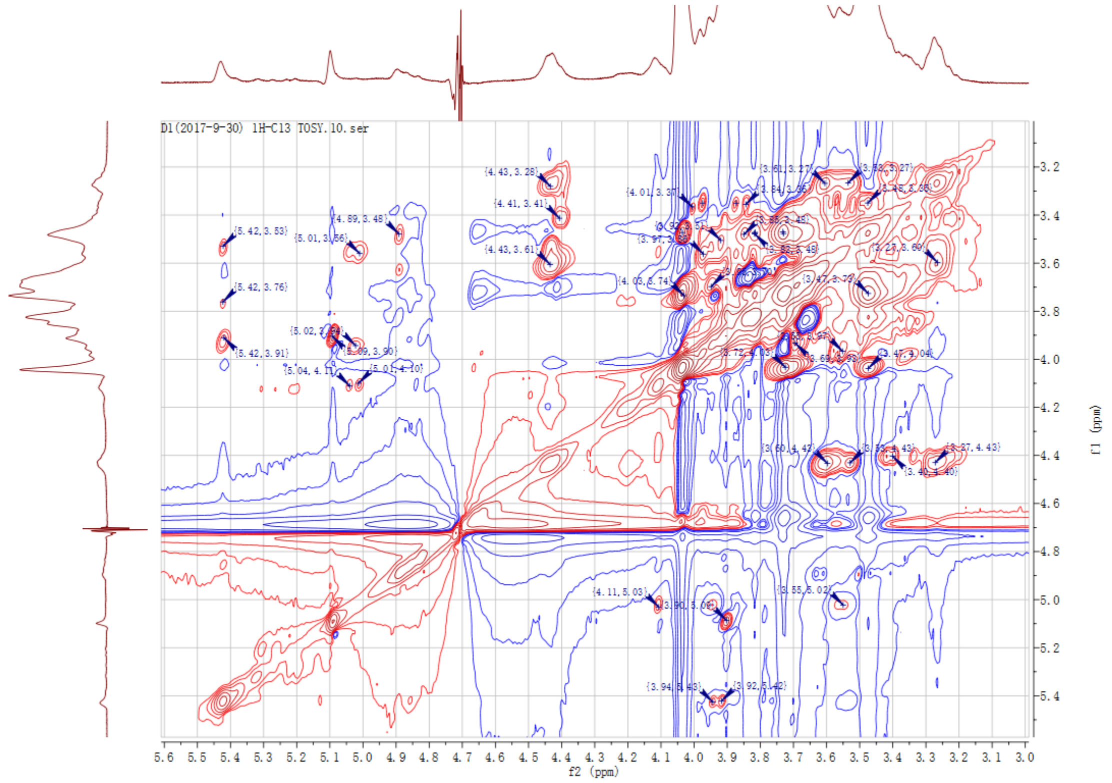


Figure S3 The TOCSY of DWDOP1

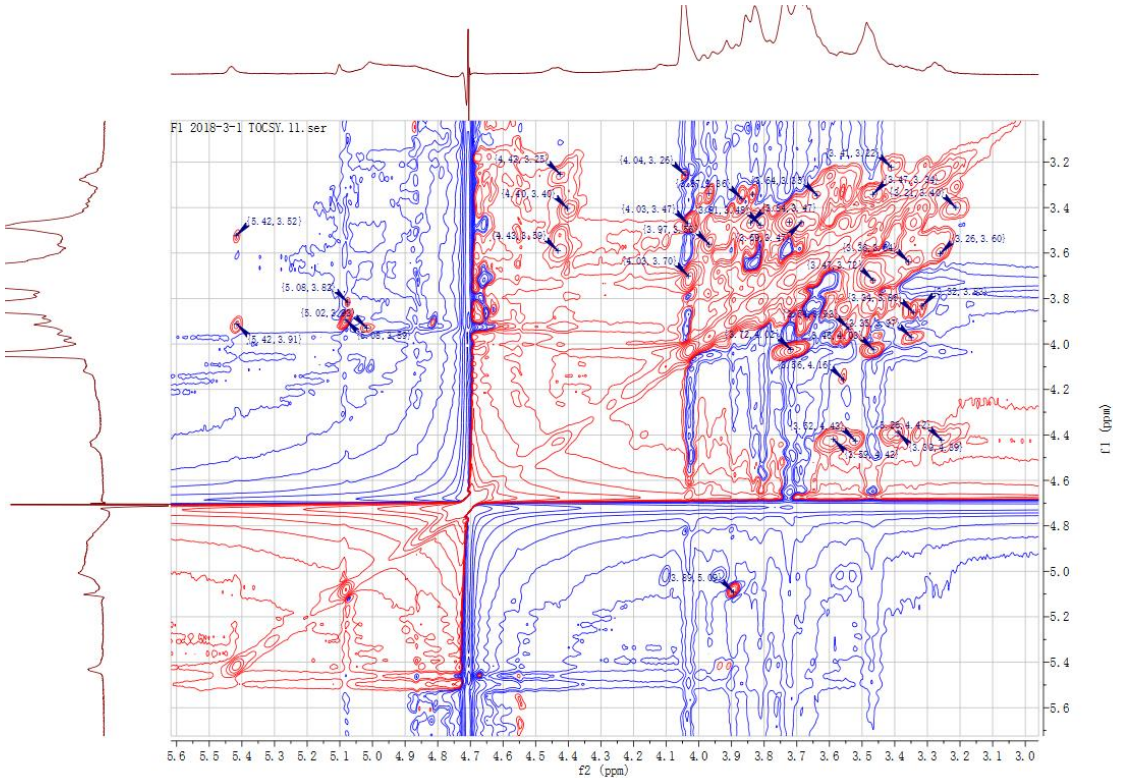


Figure 4S The TOCSY of FWDOP1

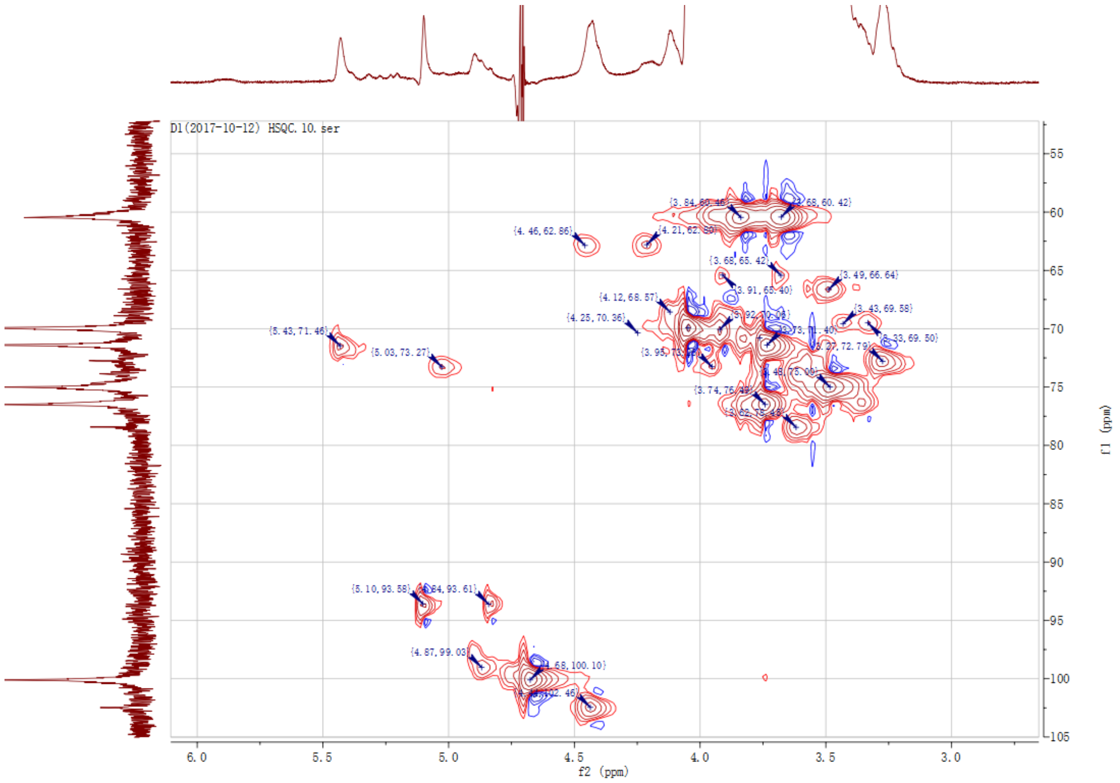


Figure 5S The $^{1}H$-$^{13}C$ HSQC of DWDOP1

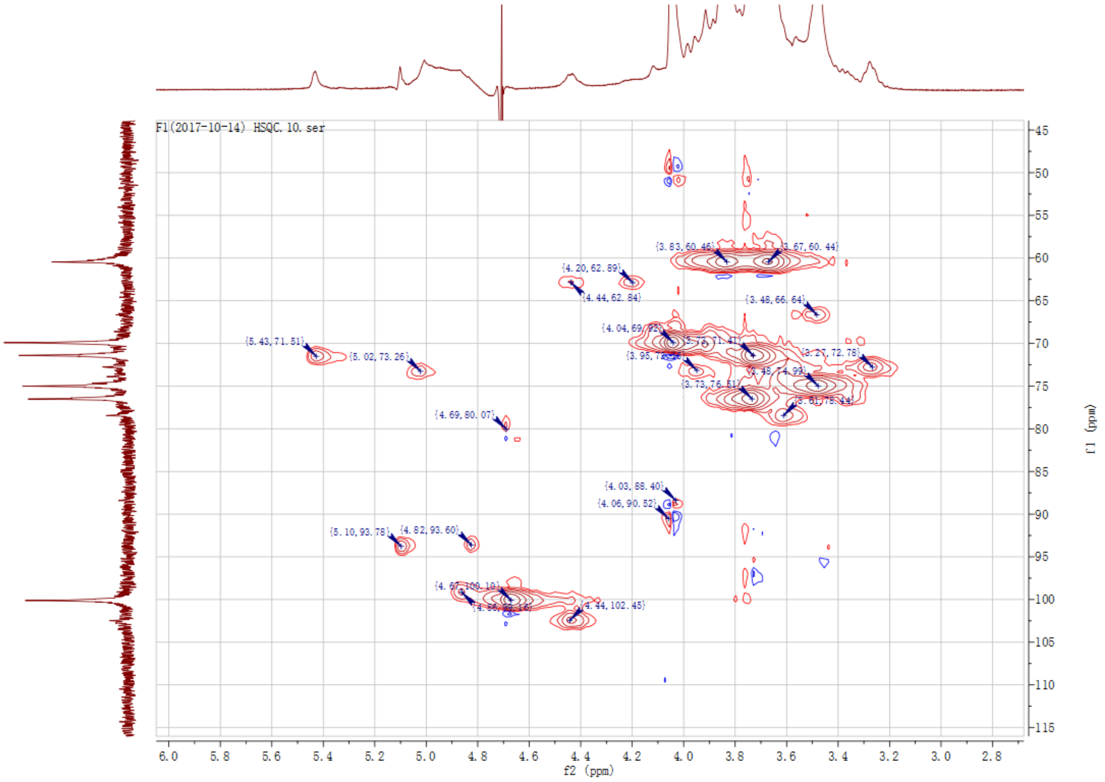


Figure 6S The $^{1}H$-$^{13}C$ HSQC of FWDOP1

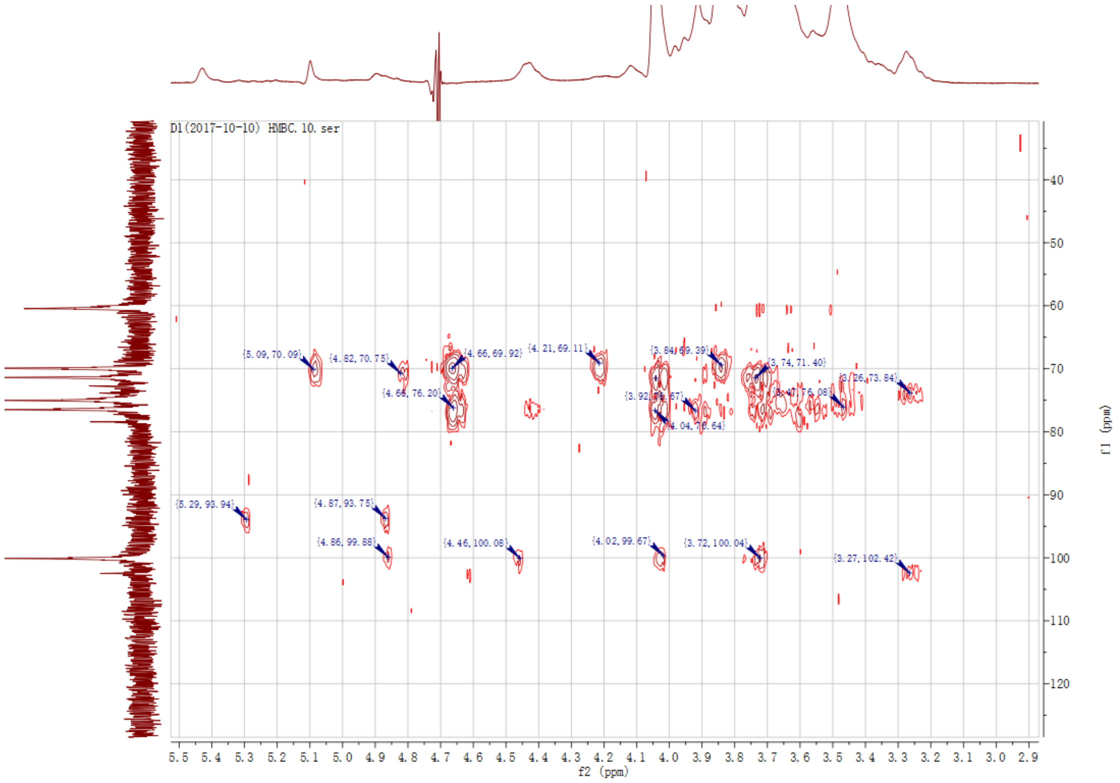


Figure 7S The $^{1}H$-$^{13}C$ HMBC of DWDOP1

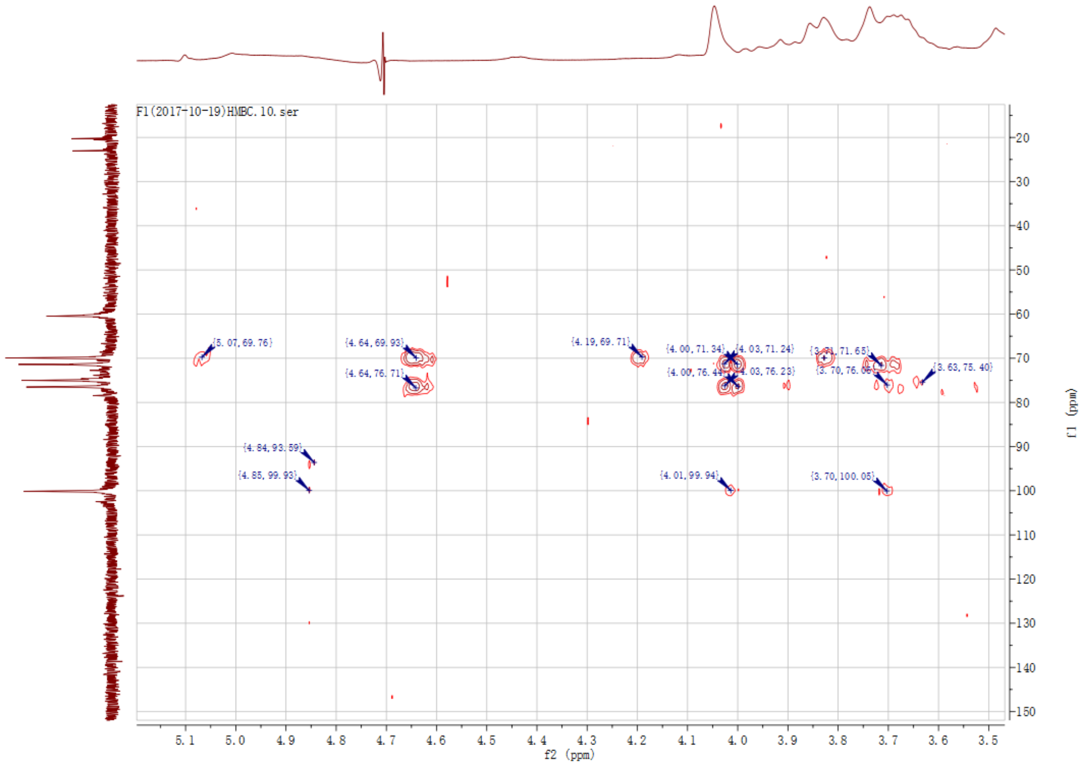


Figure 8S The $^{1}H$-$^{13}C$ HMBC of FWDOP1
